# Diet assessment of two land planarian species using high-throughput sequencing data

**DOI:** 10.1038/s41598-019-44952-3

**Published:** 2019-06-18

**Authors:** Cristian Cuevas-Caballé, Marta Riutort, Marta Álvarez-Presas

**Affiliations:** 1 Departament de Genètica, Microbiologia i Estadística, Facultat de Biologia, Universitat de Barcelona, Barcelona, Spain; 2 Institut de Recerca de la Biodiversitat (IRBio), Universitat de Barcelona, Barcelona, Spain

**Keywords:** Biodiversity, Molecular ecology

## Abstract

Geoplanidae (Platyhelminthes: Tricladida) feed on soil invertebrates. Observations of their predatory behavior in nature are scarce, and most of the information has been obtained from food preference experiments. Although these experiments are based on a wide variety of prey, this catalog is often far from being representative of the fauna present in the natural habitat of planarians. As some geoplanid species have recently become invasive, obtaining accurate knowledge about their feeding habits is crucial for the development of plans to control and prevent their expansion. Using high throughput sequencing data, we perform a metagenomic analysis to identify the *in situ* diet of two endemic and codistributed species of geoplanids from the Brazilian Atlantic Forest: *Imbira marcusi* and *Cephaloflexa bergi*. We have tested four different methods of taxonomic assignment and find that phylogenetic-based assignment methods outperform those based on similarity. The results show that the diet of *I. marcusi* is restricted to earthworms, whereas *C. bergi* preys on spiders, harvestmen, woodlice, grasshoppers, Hymenoptera, Lepidoptera and possibly other geoplanids. Furthermore, both species change their feeding habits among the different sample locations. In conclusion, the integration of metagenomics with phylogenetics should be considered when establishing studies on the feeding habits of invertebrates.

## Introduction

Land planarians (Platyhelminthes: Tricladida: Geoplanidae) inhabit moist soils around the world, with high richness levels in tropical and subtropical forests^1^. They are probably the most diverse group within the order Tricladida, containing more than 800 species^2^. They are generally small (but can reach lengths of 20 cm or more) and have nocturnal behavior, remaining hidden under rocks, litter or rotting logs during the day^3^. Although geoplanids have become a subject of interest for many researchers in recent years, there is a lack of comprehensive knowledge about their biology^4,5^.

Regarding their diet, most information comes from sporadic observations in the field. However, as many species of land planarians have recently become invasive species^6^, obtaining knowledge about their feeding habits is now crucial for the development of plans to control and prevent their expansion and to evaluate their impact in their new habitats^7–9^. This knowledge is especially important because land planarians are considered top predators within their habitat, as they feed on a wide range of soil invertebrates (including other geoplanids) and are only eaten by a limited number of species^1^.

Studying terrestrial invertebrate diets using traditional methods is hard and not that effective^10^. Both field observations of their predatory behavior and dissections to examine their digestive content require a considerable amount of time and effort given the little information these actions provide. Therefore, the vast majority of studies on the feeding habits of Geoplanidae are based on food preference experiments conducted under laboratory conditions, from the oldest^11^ to the most recent^12,13^. The methodology is simple; scientists prepare an assortment of prey that is offered one by one to the planarians, and they take notes when they attack or eat the prey. After all the prey have been offered, statistical analyses provide a general view of which prey are preferred. These studies offer a variety of prey based on high taxonomic ranks, typically class or order. The menus consist of one or two different taxa (identified or not) of earthworms (Oligochaeta), snails or slugs (Gastropoda), woodlice (Isopoda), etc. This protocol supposes an omission of any potential interactions that may occur at lower taxonomic ranks, and only changing the offered species, even maintaining their class or order rank, could lead to completely different results^14^. Conclusions related to the feeding preferences collected in these experiments are only reliable in relation to the offered catalog of prey. Because the taxonomic data of the prey catalog show often a lack of accuracy, most of these studies cannot be replicated^15^. Moreover, in animal behavior studies, laboratory conditions introduce a bias themselves^16^, as it is impossible to exactly reproduce an organism’s natural habitat. Another limitation of these experiments is that the offered prey species often do not occur in the natural habitat of geoplanids, and this limitation also has implications for the results, primarily affecting planarians with a more specific diet. Finally, the fact that most of these experiments provide a single choice, meaning that prey are offered to the same individual every few hours, implies that the moment of the offer, the sequence of previous offers and individual preferences of the specimens can distort the results.

These shortcomings can be overcome by adopting a molecular approach^17^. Early molecular prey identification was based on protein electrophoresis and immunoassays using polyclonal antisera or monoclonal antibodies^18^. Advances in DNA sequencing methods, the fast growth of global genetic databases and the emergence of bioinformatics allow the application of DNA-based methods, such as dietary analyses^19,20^, whereas many years ago, this approach was unviable.

**Figure 1:**
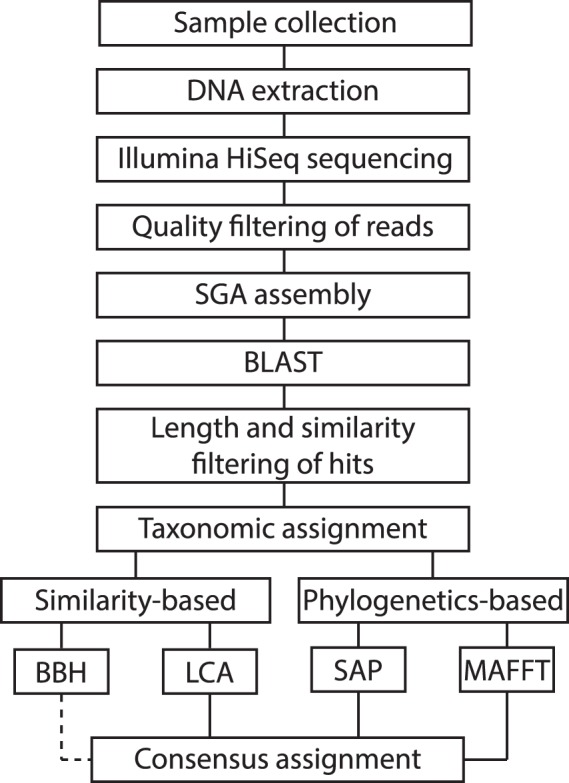
Diagram summarizing the workflow of this study from sampling to taxonomic assignment. The BBH line is broken because this method is not part of the determination of the consensus assignments.

In the present work, we take advantage of the data we previously generated using NGS from two Geoplanidae species with the goal of obtaining new molecular markers that could be used for phylogenetic and phylogeographic studies. The selected species are *Imbira marcusi*^21^ and *Cephaloflexa bergi*^22^, two codistributed land planarian species endemic to the Brazilian Atlantic Forest that have been used as model organisms in previous phylogeographic studies^23,24^. However, among the geoplanid sequences obtained with Illumina sequencing, sequences belonging to their digestive content were also obtained. Planarians feed by ingesting fluids and small pieces of tissue through peristaltic action; thus, while the initial process of digestion is extracellular, the final process is intracellular, so small pieces of the prey can remain inside the animal for long periods^25^. As a consequence, there is a fairly high possibility of extracting the DNA from the intestinal content together with the DNA of the planarian. Hence, we have recycled the “contaminating” sequences to answer an ecological question: what is the diet preference of these two species in nature? With this aim, we have developed a standardized methodology to recycle NGS genomic data to perform diet assessment analyses. We have applied four different taxonomic assignment methods (Fig. 1) based either on similarity or on phylogenetic principles that allow us to benchmark their performance when applied to the same data. This methodology favors going a step further in identifying the feeding habits of *I. marcusi* and *C. bergi*, reaching when possible, the genus or species taxonomic rank of their prey. As there are different sampled localities (see Material and Methods) for each species, we examine differences in feeding habits both between species and between populations within species.

## Results

Sequencing resulted in 41.59 Gbp and 411,789,272 reads, of which a total of 261,874 scaffolds were assembled. The results of the filtering pipeline are summarized in Table 1. The number of sequences decreased at each step for all datasets. Globally, 241 sequences remained out of 261,874 after filtering. Thus, we eliminated 99.91% of the initial sequences. The first step was the most severe, removing 98.44% of the sequences. The second step removed 93.79% of its entry pool, while the third and last steps were less severe, removing only 4.74% of its entry pool. The mean length of the sequences of the final dataset was 18% longer than the initial length of the dataset (Supplementary Table S1). The *I. marcusi* datasets had fewer sequences than the *C. bergi* datasets: ImSantoA (13), ImCubatao (23), CbSantoA (37), CbCantareira (38), CbItatiaia (47) and CbCubatao (83).

**Table 1:** Table summarizing the filtering pipeline from left to right.

	Scaffolds	Sequences that make hit in BLAST	Sequences after 95*200 filtering	Sequences after the removal
ImSantoA	19255	230	13	13
ImCubatao	22897	250	23	23
CbSantoA	29445	618	38	37
CbCubatao	25759	593	86	83
CbCantareira	98590	1481	42	38
CbItatiaia	65928	902	51	47
TOTAL	261874	4074	253	241
% of removed sequences by step	—	98,44	93,79	4,74
% of remaining sequences from the start	—	1,56	0,10	0,09

Values correspond to the number of sequences that remain after each step of the filtering process.

**Figure 2:**
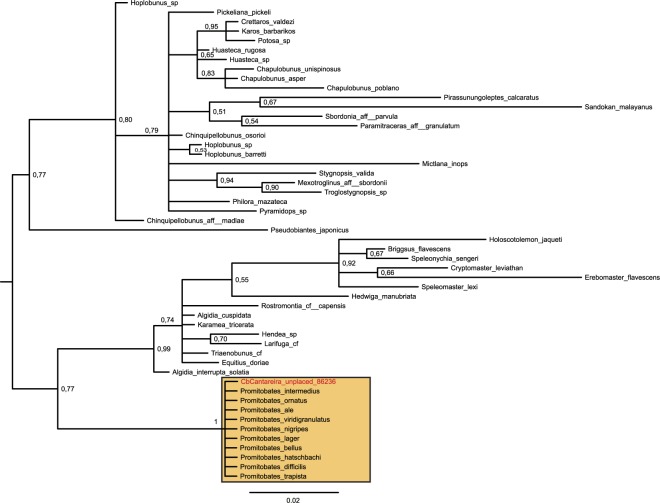
MAP tree for sequence “unplaced_86236” from CbCantareira. As the query sequence is placed in a clade with other *Promitobates* sequences with a PP = 1, MAP assigns it to the genus *Promitobates*.

For each dataset, we built a table recording the taxonomic assignment that each method made for all sequences remaining after the filtering steps (Supplementary Tables S2–S7). The Best BLAST Hit (BBH), Lowest Common Ancestor (LCA) and Statistical Assignment Package (SAP) assignments were directly retrieved from software output files, while the Molecular Assignment Pipeline (MAP) assignments came from the direct observation of the trees (e.g., Fig. 2). The lack of assignments for the SAP and MAP was due to the existence of a limited set of homologues (n < 5) that did not allow running those pipelines; missing BBH and LCA assignments were caused when two or more sequences from distinct species scored the same against the query sequence. There were several assignments to high taxonomic ranks, e.g., Bilateria, Metazoa, and Lophotrochozoa. Such assignments, often based on short sequences (<300 bp), do not allow the identification of any potential prey of the geoplanids and hence were not considered further. Finally, as expected, there were sequences assigned to *C. bergi* in all the Cb datasets and sequences that belonged to *I. marcusi* with a high probability in the Im datasets.

### Summarizing assignment results

Supplementary Tables S2–S7 show the consensus assignments of each dataset according to the criteria explained in the Material and Methods section. For instance, the sequence “unplaced_77551” from the CbCantareira dataset (Supplementary Table S7) presented these assignments: BBH to *Pachyloides thorellii* (Opiliones), LCA to Laniatores (Opiliones), SAP to Opiliones and MAP to Opiliones. As the SAP and MAP methods agreed, we considered this a consensus assignment of the sequence “unplaced_77551” to Opiliones (order). In contrast, the sequence “unplaced_6469” from the CbCubatao (Supplementary Table S5) dataset had the following assignments: BBH to the species *Sinopoda stellatops* (Araneae), LCA to RTA clade (Araneae), SAP to the species *Phonognatha graeffei* (Araneae) and MAP to Arachnida. In this case, there was no agreement between any of the LCA, SAP and MAP methods; thus, no consensus assignment was made.

**Figure 3:**
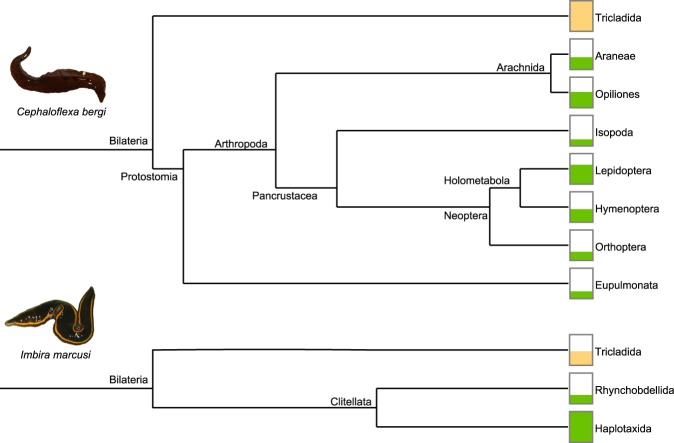
Cladograms that summarize LCA assignments at the order rank for the combination of datasets for each species. Filled bars indicate the proportion of assignments in relation to the most abundant order. Tricladida assignments (in orange) may belong either to sequenced specimens or to a preyed upon geoplanid. Photos: Fernando Carbayo.

**Figure 4:**
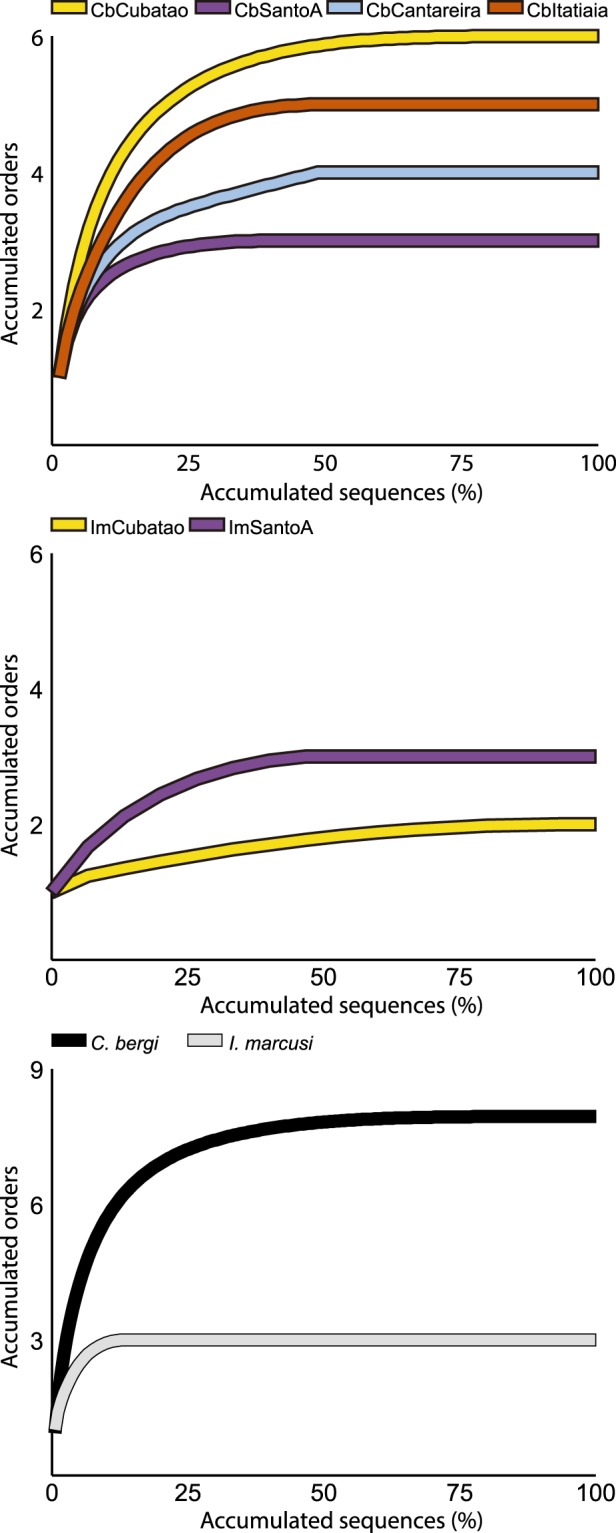
Rarefaction curves for all datasets and for whole species combinations. The increase in the number of present orders in a dataset can be observed as the sequences are incorporated.

In total, there were 138 consensus assignments out of 241 input sequences. This result indicates that a consensus assignment was made for 57.26% of the analyzed sequences. Table 2 summarizes the precision of these assignments. A total of 34.06% of the consensus assignments were made to species, 4.35% to genus and 20.29% to family, which indicates that 58.7% of the assignments were made to family or a lower taxonomic rank. Table 3 shows the results of all the nonredundant consensus assignments for each dataset. We also ran the LCA algorithm to obtain a consensus cladogram combining all the datasets for each species (Fig. 3). The cladogram shows that *C. bergi* has a more diverse diet than *I. marcusi*. Figure 4 shows the rarefaction curves for each dataset; the curves discern not only the difference between *I. marcusi* as a specialist and *C. bergi* as a generalist but also the degree of diet breadth changes between localities within a species.

**Table 2:** Number of consensus assignments by taxonomic rank for each dataset.

	ImSantoA	ImCubatao	CbSantoA	CbCubatao	CbCantareira	CbItatiaia	Total	%
Species	1	11	7	12	12	4	47	34,06
Genus	—	—	3	2	1	—	6	4,35
Family	6	4	—	4	8	6	28	20,29
Superfamily	1	—	1	1	1	2	6	4,35
Order	—	1	7	26	3	10	47	34,06
Class	—	—	—	2	—	—	2	1,45
Phylum	2	—	—	—	—	—	2	1,45
Total	10	16	18	47	25	22	138	

It is also shown the global percentage of assignments to each rank.

**Table 3:** Nonredundant consensus assignments obtained from each dataset sorted in ascending taxonomic rank.

Assignment	Rank
ImSantoA	
*Helobdella robusta* (Hirudinea)	Species
Glossoscolecidae (Oligochaeta)	Family
Lumbricidae (Oligochaeta)	Family
Geoplanoidea (Tricladida)	Superfamily
ImCubatao	
*Pontoscolex corethrurus* (Oligochaeta: Rhinodrilidae)	Species
*Pontoscolex spiralis* (Oligochaeta: Rhinodrilidae)	Species
Geoplanidae (Tricladida)	Family
CbSantoA	
*Apoecus ramelauensis* (Gastropoda: Enidae)	Species
*Cephaloflexa bergi* (Tricladida: Geoplanidae)	Species
*Imbira marcusi* (Tricladida: Geoplanidae)	Species
*Obama* sp. (Tricladida: Geoplanidae)	Genus
Hymenoptera	Order
CbCubatao	
*Caayguara albus* (Araneae: Sparassidae)	Species
*Pickeliana pickeli* (Opiliones: Stygnidae)	Species
*Hemileuca* sp. (Lepidoptera: Saturniidae)	Species
*Bombyx mori* (Lepidoptera: Bombycidae)	Species
*Trichoplusia ni* (Lepidoptera: Noctuidae)	Species
*Cephaloflexa bergi* (Tricladida: Geoplanidae)	Species
*Imbira marcusi* (Tricladida: Geoplanidae)	Species
Dugesiidae (Tricladida: Geoplanoidea)	Family
Gonyleptidae (Opiliones)	Family
Hymenoptera	Order
Isopoda	Order
CbCantareira	
*Pickeliana pickeli* (Opiliones: Stygnidae)	Species
*Cephaloflexa bergi* (Tricladida: Geoplanidae)	Species
*Promitobates* sp. (Opiliones: Gonyleptidae)	Genus
Lepidoptera	Order
Hymenoptera	Order
CbItatiaia	
*Cephaloflexa bergi* (Tricladida: Geoplanidae)	Species
Tetrigidae (Orthoptera)	Family
Opiliones	Order
Hymenoptera	Order
Lepidoptera	Order

### Comparison of method performances

**Figure 5:**
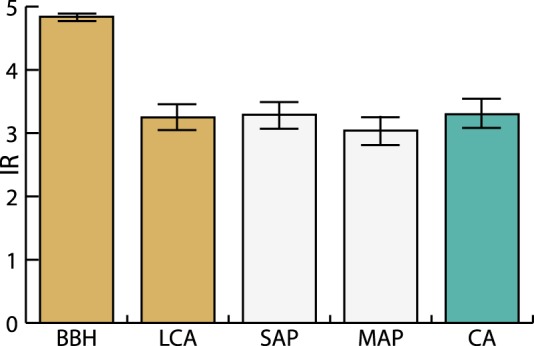
Mean Identification Resolution index (IR) and standard error for each method and for consensus assignments (CA).

We measured the success of the distinct assignment methods with two variables: the percentage of agreement that each method had in relation to the consensus assignments (Table 4) and the mean taxonomic resolution of the assignments (Fig. 5). The MAP pipeline resulted in the best method and had a 99.28% agreement with the consensus assignment, followed by the SAP pipeline, with a 90.58% agreement and LCA with a 44.93% agreement. Regarding the BBH, it had a 34.78% agreement with the consensus assignments. After the analysis of the 241 available sequences, a measure of global coincidence between pairs of methods was also computed: BBH-LCA (34%), BBH-SAP (23%), BBH-MAP (22%), LCA-SAP (22%), LCA-MAP (27%) and SAP-MAP (83%).

**Table 4:** Summary of the agreement that the distinct taxonomic assignment methods have had with the consensus assignments for each dataset and globally.

	ImSantoA	ImCubatao	CbSantoA	CbCubatao	CbCantareira	CbItatiaia	Total	% of agreement with consensus assignments
Sequences	13	23	37	83	38	47	241	—
Consensus assignments	10	16	18	47	25	22	138	—
BBH	1	11	9	10	13	4	48	34,78
LCA	3	11	11	14	16	7	62	44,93
SAP	9	16	15	43	23	19	125	90,58
MAP	10	16	17	47	25	22	137	99,28

The mean taxonomic resolution of each method was calculated with a modification of the Identification Resolution (IR) index^26^, where species assignments were given the maximum weighting (5), and as the taxonomic resolution decreased, weighting scores also decreased: genus (4), family (3), etc. (Supplementary Table S8). The IR results were BBH (4.84 ± 0.38), LCA (3.25 ± 1.57), SAP (3.29 ± 1.37), MAP (3.04 ± 1.51) and consensus assignments (3.30 ± 1.36).

### Final prey assignments

**Figure 6:**
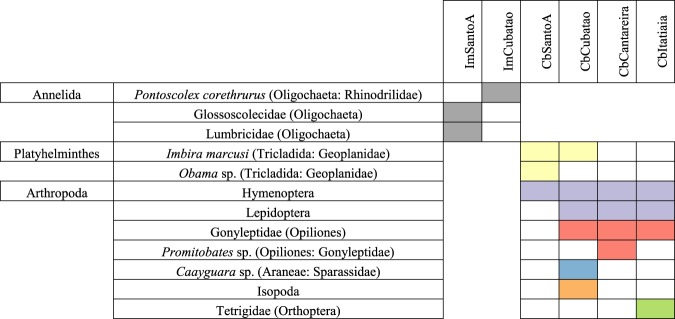
Summary of the robust assignments for all datasets and their taxonomic rank, highlighted with different colors: Oligochaeta (grey), Geoplanidae (yellow), Endopterygota (purple), Gonyleptidae (red), Sparassidae (blue), Isopoda (orange) and Tetrigidae (green).

We performed a final evaluation of the consensus and nonredundant assignments (Table 3) to discriminate the false positives. We analyzed each dataset independently and detailed the criteria and decisions undertaken to establish the final assignments (Supplementary Results) that are summarized in Fig. 6, for those below the order level. Finally, we evaluated the possibility of terrestrial planarian also being prey.

### Do the studied species prey on other land planarians?

The most polemical assignments are the ones that were unanimously assigned and include the sequences “unplaced_23142” from CbCubatao and “unplaced_20725” from CbSantoA to *I. marcusi*. Measuring 346 bp and 374 bp respectively, they match the locus 28S, which is well represented in GenBank with 247 sequences for Geoplanidae. This scenario reduces the probability of these assignments being an artifact. The trees resulting from the SAP and MAP pipelines assigned the sequences to *I. marcusi* with a PP of 1.00, and the remaining relationships were quite consistent with the phylogeny of Geoplanidae. To acquire more evidence, we inferred a MAP tree with all Geoplanidae 28S sequences in GenBank (247), where the sequences “unplaced_23142” and “unplaced_20725” are clustered to *I. marcusi* with a PP of 1 (Supplementary Fig. S1).

In the CbSantoA dataset, there were also consensus assignments to the genus *Obama*. The most robust assignment was made with a sequence of 616 nucleotides mapping onto the EF 1-alpha locus. This possible predation on *Obama* by *C. bergi* could also explain why CbSantoA is also the only dataset where we found assignments to Eupulmonata (Gastropoda). As the methodology cannot distinguish between predation and secondary predation^18,27^, Gastropoda assignments could actually be the result of a member of *Obama* (primary predator) eating a member of Gastropoda^28^ and *C. bergi* (secondary predator) preying on this *Obama* species. We consider that a plausible hypothesis because CbSantoA is the only dataset where we found assignments to both Gastropoda and to *Obama*.

## Discussion

### Method performances

Although both BBH and LCA are based on similarity, BBH-LCA agreement is poor (34%) because BBH is not stringent, and its IR is near the maximum (4.84/5). On the other hand, LCA has a conservative approach, with an IR of 3.25. The SAP and MAP agreement is high (83%) because both share a phylogenetic core with minor changes in how the assignments are made, and they also have very similar IRs (3.29 and 3.04, respectively). Table 4 shows the performance of each method in terms of coincidence with the consensus assignments; as they were determined when at least two of LCA, SAP or MAP agreed, the scores of these methods are not comparable to BBH, as the latter did not participate in determining the consensus. Thus, the agreement of the pair SAP-MAP weighed the most in determining the consensus assignments. There are no significant differences between the IR of LCA, SAP, MAP and consensus assignments, but all these values are significantly different from the BBH IR (Mann-Whitney U test, p-value ≤ 5.45^−27^), which validates our criterion of not including BBH to establish the consensus assignments. The IR of the consensus assignments (3.30 ± 1.37) is lower than in previous works that also computed an IR (~3.8 from Zarzoso-Lacoste *et al*.^26^ and 4.8 ± 0.3 from Corse *et al*.^29^) but comparable to these values if we consider that both previous works performed metabarcoding (targeting *cytochrome oxidase I*) and not metagenomics; predators were vertebrates (cats, rats and fishes), and DNA extractions were performed directly from fecal and stomach contents and not from whole individuals. The IR of the SAP method is similar between our work (3.3 ± 1.37) and that of Corse *et al*.^29^ (3.1 ± 1.1).

Generally, LCA is appropriate when sequences of the correct taxon are not represented in the database, allowing us to delimit the upper measure of confidence in polemical assignments (e.g., “unplaced_19387”, Supplementary Table S5). BBH made correct assignments only when the true taxon of the query sequence was represented in the database (e.g., “unplaced_15105”, Supplementary Table S5) but not in all such cases. For example, BBH assigns “unplaced_10078” to *Eusparassus sp*. (AN KY017360, 2180 bp, 708/725 similarity) instead of the consensus assignment to *Caayguara albus* (AN KY017358, 873 bp, 690/707 similarity), the eighth BBH. Due to its size, the largest match (AN KY017360) obtains a higher score than the shorter match (AN KY017358), even when they have the same similarity (98%), leaving the homologue representing the correct taxon lower down on the BLAST hits list^30^.

Tree-based methods (SAP and MAP) are efficient when the correct taxon is represented in the database because they are able to trace the phylogenetic signal in the sequences and cluster the query sequence with its correct taxon even when the scoring of the homologues score is fairly similar. The MAP pipeline behaved better than the SAP pipeline, even sharing the same core. Minor changes in bioinformatic pipelines could have a notable impact on the results^31^, so the difference could be due to the use of ClustalW2 or Gblocks or the fact that SAP samples 10,000 trees and MAP only 1. As the MAP pipeline agreed 99.28% of the cases with the consensus assignment, it should be the reference method when establishing future similar studies.

### Prey preferences of the studied species

Taxonomic assignment from DNA sequences has its shortcomings like any other method^32,33^. Being faster, more inexpensive and more accurate than traditional methods has its cost^34^, and this trade-off supposes that molecular barcoding can lead in many cases to erroneous assignments despite being supported by a strong theoretical background and meticulous data analysis methods^35^. After a careful examination of all the consensus assignments, we can present, in a restricted sense, a digest of the feeding habits of *I. marcusi* and *C. bergi* in this study (Fig. 6). While *I. marcusi* has a specialist diet, only feeding on earthworms and possibly on land leeches, *C. bergi* is a generalist, preying on a wide range of arthropods and possibly on other geoplanids.

**Figure 7:**
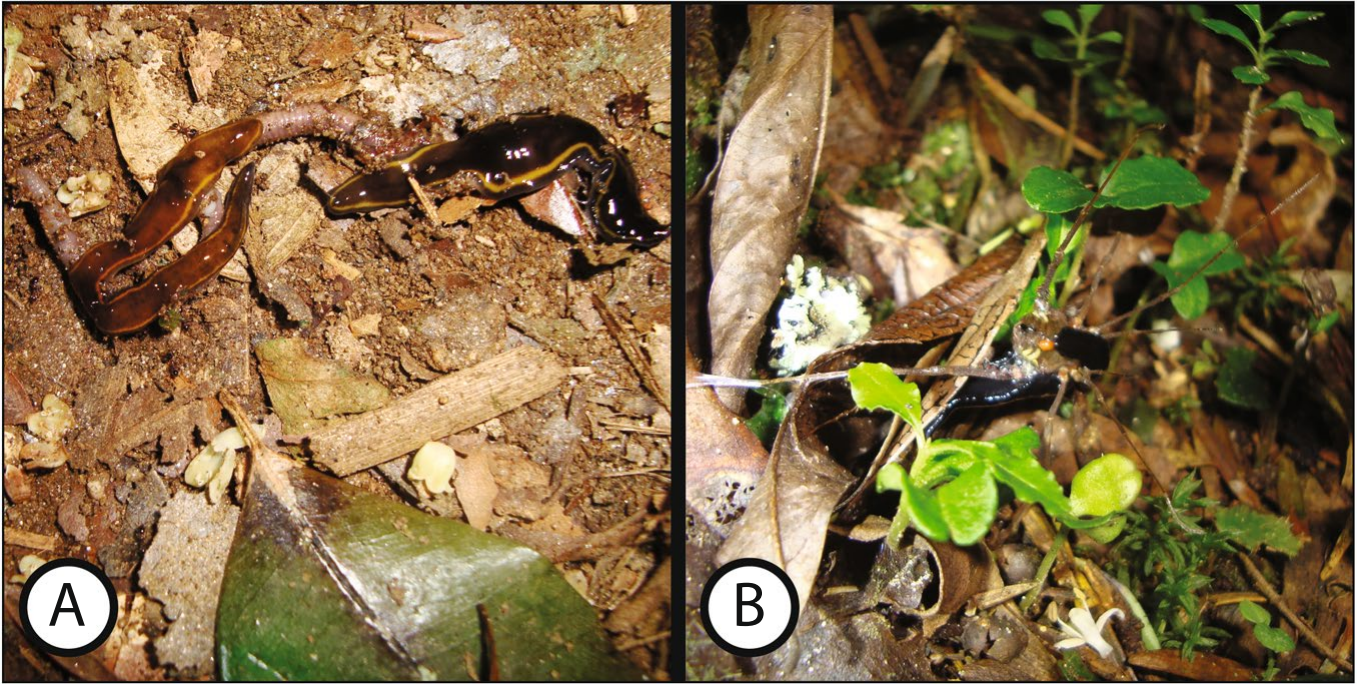
(**A**) Two specimens of *I. marcusi* eating an earthworm. (**B**) A specimen of *C. bergi* eating a harvestman. Photos: Fernando Carbayo.

In terms of *I. marcusi*, observations of its predatory behavior in nature are in agreement with our results that indicate a preference for earthworms, as two specimens of *I. marcusi* were photographed preying on an earthworm (Fig. 7A). In terms of *C. bergi*, our results are also consistent with the literature. Cseh *et al*.^13^, report observations of *C. bergi* preying on harvestmen from the family Gonyleptidae (Fig. 7B) and on an unidentified insect larva. Under laboratory conditions, the species was observed eating Orthoptera and Coleoptera^13^, and *C. bergi* was also noted as eating woodlice without details as to whether this information was from a field observation or a laboratory experimental result. The results obtained in the present work, however, provide more in-depth information than the previous results on the dietary habits of these species. The resolution of the assignments made with the presented methodology is more accurate, as we have identified not only the major taxonomic rank (class or order) of the prey but also its family, genus or species. Furthermore, we identified new feeding habits of *C. bergi* not previously detected. According to our results, obtained with a molecular approach, *C. bergi* preys on spiders (Sparassidae), harvestmen (Gonyleptidae), woodlice (*Isopoda*), pygmy grasshoppers (Tetrigidae), Lepidoptera, and Hymenoptera and could also be preying on other land planarians (Geoplanidae). In contrast, previous experiments assessing the diet of *C. bergi*^13^ only reported harvestmen (Gonyleptidae), crickets (Orthoptera) and Coleoptera larva as a part of its diet. Among the analyzed sequences, there was not one species belonging to the order Coleoptera, which highlights the importance to complement feeding trials (where organisms may eat prey that they actually do not have access to in their habitats) with molecular techniques and field observations to determine the real feeding habits of a species.

As we did not sequence a single specimen but a collection of them for each locality, our results are sensitive to the fact that the number of individuals sequenced is not the same in all localities. For instance, the locality where *C. bergi* has a broader diet coincides with the dataset where more specimens were used for the DNA extraction. We sequenced a total of 19 specimens, 7 of *I. marcusi* and 12 of *C. bergi*. Therefore, the results presenting *C. bergi* as a generalist and *I. marcusi* as a specialist may be due in part to these differences in sample size, as our methodology is not entirely interpretable as being quantitative. Nonetheless, if *C. bergi* is a specialist, then the number of different taxonomical assignments would not increase when using more specimens for the sequencing, and this was not the case (Fig. 4). Moreover, the fact that in the locality with a similar number of individuals (Cubatao, 5 for Cb and 4 for Im), we found the two extremes with Cb having the maximum diversity of prey and Im having only one prey species, supporting our conclusion that *C. bergi* is a generalist and *I. marcusi* is a specialist.

Prey variability among localities can also be influenced by the matrix heterogeneity, and *C. bergi* shows a conserved prey preference for harvestmen (Cubatao, Cantareira and Itatiaia), Hymenoptera (SantoA, Cubatao, Cantareira and Itatiaia) and Lepidoptera (Cubatao, Cantareira and Itatiaia). Other prey, such as geoplanids (SantoA and Cubatao), spiders and Isopoda (Cubatao) or grasshoppers (Itatiaia), are more restricted. However, the lack of exclusive prey in SantoA and Cantareira can be a consequence of those two localities being the ones with fewest sequenced individuals (2). In SantoA, the most commonly preyed upon taxa (harvestmen and Lepidoptera) did not appear. As land planarians have a limited dispersal capacity^36^, events such as habitat fragmentation may lead to the formation of land planarian communities with heterogeneous structures within the same biotope^37^. *C. bergi*, as a generalist, could be feeding on the niche with the least competition it has access to in each locality. The shared habit of preying on other geoplanids between SantoA and Cubatao may be attributable to the fact that these localities are the closest ones among those that were sampled (25 km apart), so their habitats would be presumably similar.

*I. marcusi* preys on *Pontoscolex corethrurus* (Rhinodrilidae) in Cubatao and on Lumbricidae and Glossoscolecidae in SantoA. The Cubatao locality is in the lowlands (40 m a.s.l.) and close to the city of Cubatão (127,000 inhabitants), so we can consider it more degraded than the SantoA locality (931 m a.s.l.), which is quite isolated from the closest cities. As *P. corethrurus* is more abundant in degraded habitats^38^, the presence of this earthworm in Cubatao could be overwhelming as it is replacing endemic earthworm species of the Brazilian Atlantic Forest^39^, while in SantoA, *I. marcusi* would still be preying on endemic earthworms.

Briefly, the identified feeding behavior within a species is a glimpse of its overall diet and is affected by methodological artifacts, such as sampling bias and by biological factors, such as competition or matrix heterogeneity. However, how distinct food preferences have evolved among land planarian species is unclear. A recent compilation of Geoplaninae predatory behavior suggests that closely related species of geoplaninids tend to have similar diets^13^. The genera *Cephaloflexa* and *Choeradoplana* (phylogenetically sister groups) have cephalic specializations that are intimately related to the capture of harvestmen^40^. However, even within a genus, we found diet variability. Species of *Obama* feed prominently on land gastropods but on different species^12^, which allows them to coexist with a minimum niche overlap. This niche differentiation can arise via behavioral plasticity or via evolutionary shifts in genetic variance^41^. We can see the predatory behavior as a rugged fitness landscape with demes exploring and exploding the genotype space towards a local optimum, where a different behavior would be the result of demes settled at different optimums. Ecological generalists are often nothing but a heterogeneous collection of relatively polymorphic individuals^42^. Such among-individual variation is an important target for natural selection, promoting diversification favoring rare (and thus fittest) types. This diet diversification from a generalist ancestor could result in speciation^43^ even in the presence of gene flow as hybrids would fall between foraging niches, leading to postmating reproductive isolation. We suggest that in geoplanids, diet could have acted as a “magic trait” having a large influence on speciation^44–46^. Predatory behavior would have been under strong divergence selection, increasing resource usage, reducing competition and allowing different species to coexist in the same habitat.

### Conclusions and final remarks

The application of the presented methodology can be especially interesting in the case of assessing whether a land planarian has the potential to become an invasive species or not^47^, as a broad dietary spectrum is often a characteristic trait of invasive species^48^. The ease with which these data can be collected and analyzed makes this methodology suitable for application in the early stages of species invasion^49^, as we only need a few specimens. The lack of information on invasive species is such that there are Brazilian species that have been newly described from specimens found in Europe and not in Brazil^50^. Evaluating our results, *C. bergi* would have the potential to become an invasive species, as it has a generalist diet and shows high variability between localities. By contrast, *I. marcusi* has a restricted diet, and its dietary changes between localities could be due to local prey availability rather than plasticity. Nonetheless, because having a wide dietary spectrum is only one of the traits that characterizes invasive species, we cannot fully assess the invasive potential of the studied species. On the other hand, knowledge of the potential diet of these species highlights the greater danger of a possible invasion of *I. marcusi* or a species with a similar diet, given that a direct increase in predation on earthworms could have a greater economic impact in agricultural regions, where the lack of this animal could affect the crops in terms of the quality and health of the soil^51,52^.

The combination of metagenomics with phylogenetic assignment methods presented in this work succeeded in elucidating the *in situ* diet of invertebrates, whereas previous metabarcoding^53–55^ or metagenomic^56,57^ diet assessment studies were largely based on vertebrates. Molecular-based diet studies of invertebrates are limited and often based only on feeding trials^58,59^. After Paula *et al*.^60^, this study is, to our knowledge, the second metagenomic work that identifies the *in situ* diet of an invertivorous invertebrate. In addition, this study yielded a high dietary resolution, as we have assigned sequences to endemic Brazilian spiders and harvestmen with a very limited distribution range. One of the shortcomings of not generating NGS data but instead recycling them is that we have only used a small amount of the original data. This is because NGS technologies generate a high number of reads, which has the potential to magnify the effect of erroneous sequences due to the low quantity and quality of the eDNA, chimeras and contamination^61^. Sequencing only the digestive content and not the whole individual could also help to improve the quality of the retrieved data because less predator DNA would be present in the extracted samples.

## Material and Methods

### Sampling localities

**Figure 8:**
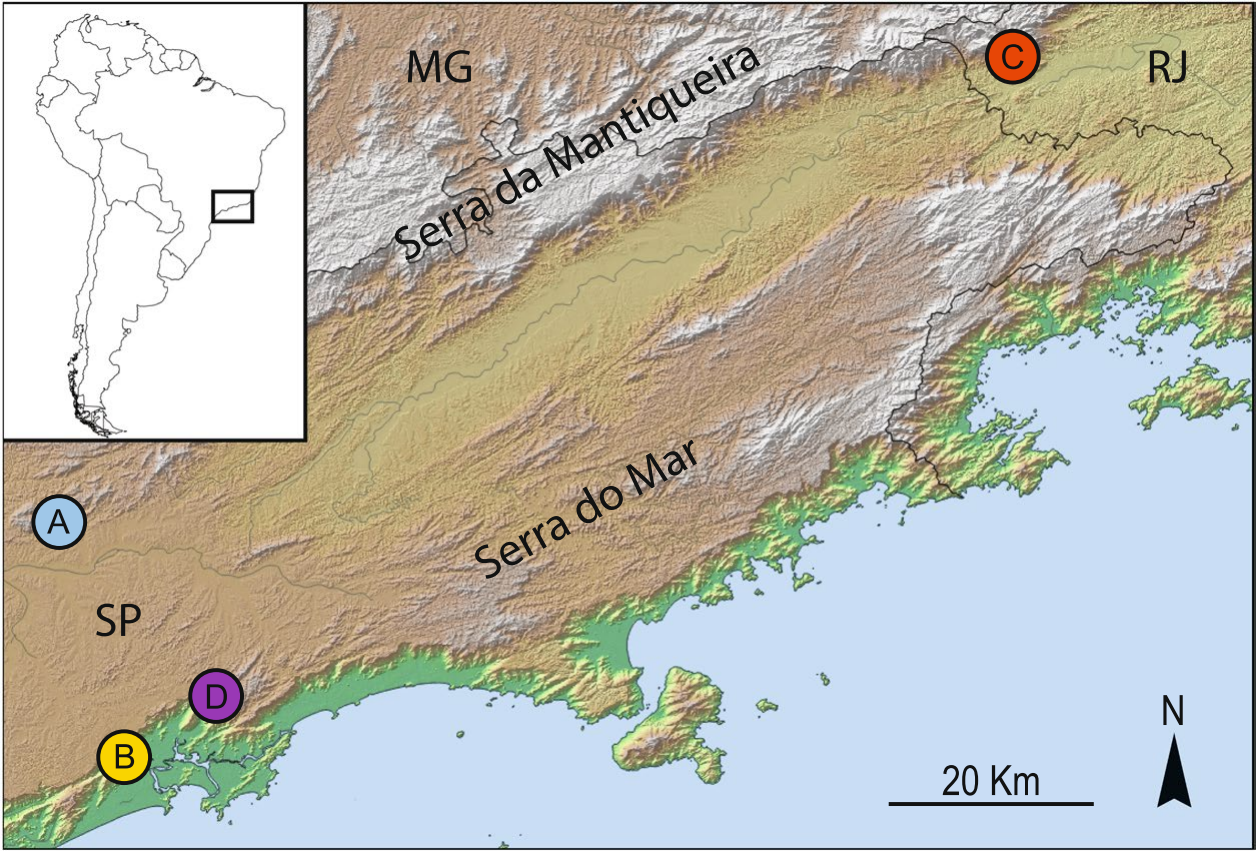
Sampling localities of the specimens used in this study: Cantareira (**A**), Cubatao (**B**), Itatiaia (**C**) and SantoA (**D**). The map spans the states of São Paulo (SP), Minas Gerais (MG) and Rio de Janeiro (RJ).

The planarians were collected by Dr. Fernando Carbayo’s group in a field campaign in Brazil between April and July 2009. After their collection, specimens were dipped in boiling water and immediately fixed in absolute ethanol. Thus, planarians were not able to completely digest the most recent prey on which they had fed. Samples were collected from the following four different localities (Fig. 8): A: Parque Estadual da Serra da Cantareira, city and state of São Paulo (−23.42914, −46.6325; 1075 m a.s.l.); B: Parque Estadual da Serra do Mar: Núcleo Itutinga Pilões, city of Cubatão, state of São Paulo (−23.90778, −46.4892; 40 m a.s.l.); C: Reserva Biológica do Alto da Serra de Paranapiacaba, city of Santo André, state of São Paulo (−23.76907, −46.2855, 931 m a.s.l.); and D: Parque Nacional de Itatiaia, city of Resende, state of Rio de Janeiro (−22.45100, −44.6082; 839 m a.s.l.). All sampling points are located within conservation areas of the Atlantic Forest ecoregion, one of the most important biodiversity hotspots in the world^62^. Specifically, they all belong to the Serra do Mar coastal forest biome, and except for B, which is in the lowlands, the remaining locations are situated in montane stage.

The localities are referred to as the following: Cantareira (Parque Estadual da Cantareira), Cubatao (Parque Estadual da Serra do Mar), SantoA (Reserva Biológica do Alto da Serra de Paranapiacaba) and Itatiaia (Parque Nacional de Itatiaia). For *C. bergi*, we have samples of specimens from all four localities, while for *I. marcusi*, samples are only available from Cubatao and SantoA. Each of these six combinations of species-locality constitutes a dataset, as we analyzed them separately. The datasets are named according to which species (Im for *I. marcusi* and Cb for *C. bergi*) and locality they represent.

### DNA extraction, sequencing and assembly

A high-quality DNA extraction was performed combining various specimens per locality as follows: ImCubatao (4), ImSantoA (3), CbCubatao (5), CbSantoA (2), CbItatiaia (3) and CbCantareira (2). To perform the DNA extraction, a Promega lysis buffer was used in combination with 10 µL of proteinase K at 20 mg/mL, followed by a traditional extraction using a phenol-chloroform protocol^63^ with some modifications. The extracts were incubated with 15 µL of RNase A at 10 mg/mL for two hours at 37 °C. The DNA was quantified with an Invitrogen Qubit Fluorometer 2.0 (Broad Range kit). A paired-end library was prepared with Illumina TruSeq DNA Library Prep kit (400 bp insert size) and run in an Illumina HiSeq 2000 (2 × 100 bp) sequencing device by Macrogen (Korea).

Prior to these analyses, the raw data were quality filtered with NGS QC Toolkit^64^; parameters were a cut-off read length for HQ of 70% and a cut-off quality score of 20. The string graph assembler (SGA)^65^ assembly was performed under the following parameters: overlap (75), k-mer correction (41), coverage filter (2), fm-merge overlap (55), small resolution (10), minimum pairs (5), minimum length (200), scaffold tolerance (1) and maximum gap difference (0).

### Filtering process

The main purpose of generating the NGS data was to obtain molecular markers from the genomes of *I. marcusi* and *C. bergi*, not to study their diets. Thus, we could not proceed to the application of the taxonomic assignment methods without first performing adequate filtering. To analyze such a volume of information demands a great amount of time and computing power, and because the bulk of sequences will not contribute any information, the analysis of the entire dataset would not be useful. Therefore, we designed a filtering pipeline to retain only the most informative sequences. Furthermore, reducing the amount of data processed allowed us to trace the sequences through the future analysis pipeline and supervise, if needed, each case manually. This filtering pipeline was structured in three steps and outlined in the following paragraphs: 1) remove the sequences that do not match any others represented in the database, 2) maintain only the most informative sequences and 3) purge conflicting sequences.

For step 1, a BLAST^66^ search was performed for each of the six datasets: ImCubatao, ImSantoA, CbCubatao, CbSantoA, CbCantareira and CbItatiaia. This search was conducted against the GenBank^67^ nonredundant nucleotide database, which we downloaded as local. With the BLAST + 2.6.0^68^ command-line tool, an expected value of 0.0001 was used to perform the search with parameters -task megablast and -max_target_seqs 100. The results were downloaded in tabular format. Then, we obtained the names of the sequences that had at least one BLAST hit in the search. Given a vector with such names, the original data were parsed with the pyfaidx Python module^69^, which we also used for the following parsing steps.

The next phase of filtering was consistent with the need to keep only the most informative sequences, which are the ones with enough information to allow us to determine taxonomic assignments to ranks such as family, genus or species. We set the threshold for keeping a sequence with at least one BLAST hit longer than 200 nucleotides at the 95% or higher level of similarity. This threshold was determined after some fine-tuning, with softer thresholds leading to flagrant false positives and misleading assignments.

In the final step, we manually removed conflicting sequences, such as cloning vectors, suicide vectors or sequences with annotation problems in the database.

Because our goal was to assess the diet of the planarians, we could have also removed all the sequences suspected of belonging to *I. marcusi* and *C. bergi* to simplify the datasets. However, we did not do this for two reasons. First, it is well known that some land planarians prey on other geoplanids^3^. Second, and more importantly, we used the sequences suspected of belonging to the sequenced specimens as a positive control of our methodology. As land planarians belong to the order Tricladida and the NGS data that we recycled came from DNA extractions from whole specimens, the lack of Tricladida assignments in the results would be a sign that our methodologies failed. In contrast, the presence of Tricladida assignments in the results, particularly assignments to *I. marcusi* in Im datasets and to *C. bergi* in Cb datasets, would certify that both the filtering and the analysis pipelines worked properly. Moreover, we determined whether these assignments came from the sequencing of the specimen itself or the sequencing of a prey item.

### Taxonomic assignment

The assignments were conducted from the perspective of four different methods (Fig. 1): two based on similarity (Best BLAST Hit (BBH) and Lowest Common Ancestor (LCA)) and two based on phylogenetics (Statistical Assignment Package^30^ (SAP) and Molecular Assignment Pipeline (MAP)). Having four different assignments for each sequence allowed us to both benchmark the behavior of the methods when applied to the same data and make a consensus assignment for each sequence while accounting for the peculiarities of each method.

The BBH method consists of assigning the sequence to the same taxonomic rank of its best BLAST hit in GenBank, or if there is more than one hit with the same score, then the assignment is to their lowest common taxonomic rank. For every single sequence, we manually noted which was the BBH by conducting a search via Web BLAST. The most commonly used method in metabarcoding is BLAST, but it has some caveats: 1) mapping scores are based on local (not global) alignments, 2) it completely ignores population genetics and phylogenetic issues, and 3) it does not provide measures of confidence for the taxonomic assignments^30^. Despite all these shortcomings, we decided to consider BLAST, as it is fast, and because as the most used, it was important to compare it to other methodologies.

**Figure 9:**
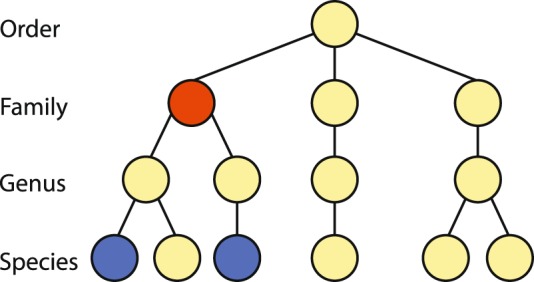
Given a selection of sequence topper BLAST hits (in blue, similarity ≥95%), the lowest common ancestor (LCA) algorithm quickly computes their LCA (in red).

The LCA method also uses BLAST searches but combines the searches with an LCA algorithm that makes the method quite effective. These algorithms (Fig. 9) find the lowest common taxonomic rank among the selected BLAST hits from a sequence, so although they are fast, they are not precise. BLAST was performed with the LCA algorithm implemented in the MEGAN software^70^. For each sequence, we calculated the LCA (weighted mode) of all BLAST hits that met the similarity criteria of ≥95%. We performed the LCA method for each dataset and for each species separately to detect the taxonomic dispersion of their diets. As this approach is very conservative, we considered the assignments made by this method to be our upper measure of confidence.

The SAP pipeline also uses the GenBank database. After performing a BLAST search, the program compiles a set of homologues for each sequence to maximize the taxonomical dispersion of this set. Then, these homologues (up to 50 when possible) and the query sequence are aligned with ClustalW2 2.0.8^71^. Next, 10,000 trees are sampled from the Markov chain Monte Carlo (MCMC) analysis performed using MrBayes^72^. These trees map the taxonomic annotation of each clade (the lowest taxonomic rank that includes all the sequences of that clade) onto their nodes, so the taxonomic rank of the sister clade to the sample sequence is identified. Finally, the posterior probability (PP) of the query sequence forming a monophyletic group with a given taxon is calculated as the fraction of sampled trees where the sister clade to the sample sequence is a member of that taxon. Assignments were made only at a PP ≥0.95.

Because Clustal has been nowadays overcome by other alignment programs^73,74^, we opted for designing a parallel pipeline maintaining the SAP philosophy using MAFFT 7.310^75^ to align the sequences. The MAP jumps from the compilation of the homologues directly to MAFFT 7.310 instead of going to ClustalW2. After the G-INS-i alignment was performed, it was processed via Gblocks 0.91b^76,77^ to remove poorly aligned positions. The Gblocks parameters were the minimum length of a block (5), allowed gap positions (with half) and maximum number of contiguous nonconserved positions (10). Next, a phylogenetic tree was constructed with MrBayes 3.2.2 (three million generations, samplefreq = 1000, burnin = 0.25, nruns = 2) remotely on CIPRES Science Gateway V. 3.3^78^. Finally, each tree was visualized with FigTree 1.4.2^79^. Rooting the tree at the midpoint, the taxonomic assignment was made to the LCA of the most terminal clade where the query sequence belonged with a PP ≥0.95.

The taxonomic assignments of all four methods were recorded and compared for each sequence. When at least two methods among LCA, SAP and MAP made the same taxonomic assignment, it was considered to be a consensus assignment. When two or more consensus assignments to the same taxon were present within a dataset, they were considered redundant assignments of the taxon. Only consensus and nonredundant assignments were analyzed. However, common phenomena such as incomplete lineage sorting, incomplete databases, incorrect annotations or precarious sequences^61,80^ could result in false positives. Therefore, we examined the consensus assignments to determine whether they were reliable based on the sequence length and the matching locus, looking mainly at whether the locality where the assignment was made was within the known distribution range of the assignment’s taxonomic rank. Individual-based rarefaction curves were computed and extrapolated with EstimateS 9.1.0^81^ for all datasets based on the order of the LCA assignments.

## Supplementary information

Supplementary Tables

Supplementary Information

## Data Availability

All the analyzed sequences (241) are deposited in the Dryad Digital Repository: 10.5061/dryad.bg1kq06.
