# Supplementary material for: Diet assessment of two land planarian species using high-throughput sequencing data
Source: Sci Rep. 2019 Jun 18;9:8679. doi: 10.1038/s41598-019-44952-3 (PMC6581950; doi:10.1038/s41598-019-44952-3)
Supplement: Supplementary file 2 — Supplementary Information [file 41598_2019_44952_MOESM2_ESM.pdf]

Supplementary Information

Diet assessment of two land planarian species using high-throughput sequencing data

Cuevas-Caballé, C[a,b]; Riutort, M[a,b]; Álvarez-Presas, M[a,b]*

[a] Departament de Genètica, Microbiologia i Estadística, Facultat de Biologia, Universitat de Barcelona, Barcelona, Spain

[b] Institut de Recerca de la Biodiversitat (IRBio), Universitat de Barcelona, Barcelona, Spain

* Corresponding author. E-mail address: onaalvarez@ub.edu

Supplementary Results

ImSantoA

The Glossoscolecidae family is native to South America and well represented in Brazil [1], while Lumbricidae is native to Europe but invasive and widely distributed in temperate regions. In Brazil, the Lumbricidae family is restricted to the southern and southeastern states [2], coinciding with the localities of the study, so we accepted these two assignments as good.

It is unlikely that the leech *Helobdella robusta* is a prey of *I. marcusi*. While the *Helobdella* genus occurs worldwide [3], it is quite improbable that a land planarian preys on this group because geoplanids avoid flooded water environments [4]. Because the *H. robusta* genome is completely sequenced as it is a model organism for annelid and lophotrochozoan development, it is overrepresented in GenBank. Moreover, the sequences assigned to *H. robusta* were annotated to hypothetical proteins from mRNA sequences, a region that is not usually sequenced outside of genomic studies. Consequently, it is probable that the assignment may actually correspond to a genomic region of an earthworm or, less probable, to a land leech.

ImCubatao

I. marcusi appears to feed on two species of *Pontoscolex* (Rhinodrilidae: Oligochaeta) in Cubatao. *Pontoscolex corethrurus* is native from the Guiana Shield region [5], but currently, the species presents a broad distribution in tropical regions and in as many as four continents and sixty countries [6]. On the other hand, *Pontoscolex spiralis* has a more restricted distribution. It appears to be originally from Puerto Rico [7,8] but has colonized the Lesser Antilles, as it has been reported in Guadeloupe and Martinique [9,10], and has

reached French Guiana [11].

The broad distribution of *P. corethrurus* and the fact that it has been cited within the Cubatão range [12] make our assignment to this species robust. However, for *P. spiralis* assignments, we must be more cautious as French Guiana is far north of this locality. Closely examining the sequences that map to 18S ("unplaced_6169", 465 bp) and 28S ("unplaced_7401", 448 bp and "unplaced_11711", 369 bp) of *P. spiralis* in GenBank, we found that these sequences map onto a region not present in the existing *P. corethrurus* 18S and 28S sequences because the latter are much shorter. These sequences probably also belong to *P. corethrurus*, which recent works suggest is actually a species complex [13]. The sequences of the individuals preyed by *I. marcusi* correspond to the *P. corethrurus* complex sp. L1, the most widespread lineage.

CbSantoA

The *Apoecus ramelauensis* assignment is somewhat odd as this species is endemic to Mount Ramelau on Timor island [14]. The sequence has 461 bp and maps onto 28S sequences in GenBank. As the 28S locus is widely used in Mollusca, it is unlikely that this exotic assignment was due to loci misrepresentation. Because there are a handful of consensus assignments to Eupulmonata (Gastropoda) in CbSantoA, we treated this assignment as Eupulmonata despite the unanimous *A. ramelauensis* assignment.

CbCubatao

The spider genus *Caayguara* is endemic to the Brazilian Atlantic Forest, with many species occurring in the state of São Paulo [15]. The matching locus is 28S, and the assigned sequences are "unplaced_15105" (371 bp) and "unplaced_10078" (749 bp). As *Caayguara albus* is the only species of the genus in GenBank, we must take this

assignment as a *Caayguara* sp. *Pickeliana pickeli* is the only species of the *Pickeliana* genus represented in GenBank. Its distribution is well known and modeled [16], and our sampling sites are out of its range. Dr. Marcio Bernardino da Silva confirmed that the Stygnidae family does not occur within the Cubatão range (personal communication). The sequence used for the assignment is rather long (780 bp), the assignment is almost unanimous, the locus is widely sequenced (28S), and *Pickeliana pickeli* assignments are present more than once in other localities. As a consequence, we consider this a controversial case; for these cases, the LCA assignment method is the most conservative, and we treated this assignment as a being in Laniatores, a suborder of Opiliones containing, among others, the Stygnidae and Gonyleptidae families. Nonetheless, as we have consensus assignments to these species coming from two different datasets, we did not discard the possibility that these sequences actually belong to a genus of the Stygnidae family.

The *Bombyx mori* assignment is made with a short sequence (272 bp) where all of the homologues also match *Bombyx mori* for the chorion locus. Given that and the fact that *Bombyx mori* is broadly represented (166927 sequences in GenBank), the most plausible hypothesis is that the query sequence belongs to a nonidentified Lepidoptera. The assignment made to *Hemileuca* sp. (352 bp) matches the 28S locus, and the *Trichoplusia ni* assignment (1335 bp) has a very limited set of homologues (7) included in the trees and matches with transposable elements. As there are many assignments to Lepidoptera in this dataset, we consider these *Hemileuca* sp. and *Trichoplusia ni* sequences as Lepidoptera, despite the LCA assignments being to Arthropoda and *Bombyx mori,* respectively.

The Dugesiidae assignment is clearly an artifact. It was made with a quite short sequence (268 bp), and the matching locus is 28S. LCA, our most conservative method, assigned

it to Continenticola, a clade that contains land and freshwater planarians. Geoplanoidea (Geoplanidae and Dugesiidae) has two types of 28S [17], but type-I is predominant in most studies. Here, a previously unsequenced type-II of the *Cephaloflexa bergi* 28S could be mapped with the well-known and represented GenBank type-II 28S of Dugesiidae.

CbCantareira

Gonyleptidae is a family of Neotropical Opiliones especially rich in the Atlantic Forest of Brazil [18]. The sequence "unplaced_86236" (525 bp) from CbCantareira is assigned to the genus *Promitobates* (Gonyleptidae) (Figure 4). This harvestmen genus is endemic to the area where all the specimens of our study were collected [19], consisting of the northern state of Santa Catarina, the states of Paraná and São Paulo and the southern state of Rio de Janeiro. Thus, this is a quite robust assignment, as we retrieved a species endemic to the sampling location of its predator from the data. The case of *Pickeliana pickeli* has already been analyzed in the CbCubatao dataset.

CbItatiaia

The Tetrigidae assignments were numerous in the CbItatiaia dataset. This family of Orthoptera, also known as pygmy grasshoppers, is cosmopolitan and especially rich in tropical forests [20], so the assignments to Tetrigidae are consistent with its distribution range.

References

1. Siqueira, F. de F. *et al.* Genetic diversity and population genetic structure in giant earthworm Rhinodrilus alatus (Annelida: Clitellata: Glossoscolecidae). *Pedobiologia (Jena).* **56,** 15–21 (2013).
2. Christoffersen, M. L. Catalogue of the Lumbricidae (Annelida, Clitellata, Lumbricoidea) from South America, with remarks on the systematics of the Lumbricina. *Zoosystema* **33,** 141–173 (2011).
3. Kutschera, U., Langguth, H., Kuo, D. H., Weisblat, D. A. & Shankland, M. Description of a new leech species from North America, Helobdella austinensis n. sp. (Hirudinea: Glossiphoniidae), with observations on its feeding behaviour. *Zoosystematics Evol.* **89,** 239–246 (2013).
4. Froehlich. On the Biology of Land Planarians. *Bol. da Fac. Filos. Ciencias e Let. da Univ. Sao Paulo* 263–272 (1955).
5. Righi, G. On a Collection of Neotropical Megadrili Oligochaeta II. Glossoscolecidae, Lumbricidae. *Stud. Neotrop. Fauna Environ.* **19,** 73–87 (1984).
6. Ortiz-Gamino, D., Pérez-Rodríguez, P. & Ortiz-Ceballos, A. I. Invasion of the tropical earthworm *Pontoscolex corethrurus* (Rhinodrilidae, Oligochaeta) in temperate grasslands. *PeerJ* **4,** e2572 (2016).
7. González, G. *et al.* Earthworm communities along an elevation gradient in Northeastern Puerto Rico. *Eur. J. Soil Biol.* **43,** (2007).
8. Lugo, A. E. *et al.* Preliminary estimate of earthworm abundance and species richness in Spathodea campanulata Beauv. Forests in northern Puerto Rico. *Caribb. J. Sci.* **42,** 325–330 (2006).

9. Fragoso, C., Kanyonyo, J., Moreno, A., Senapati, B. K. & Blanchart, E. A Survey of Tropical Earthworms : Taxonomy , Biogeography and Environmental Plasticity. *Earthworm Manag. Trop. agroecosystems* **CAB intern,** 1–26 (1999).

10. James, S. W. & Gamiette, F. New species of Dichogaster Beddard, 1888 (Clitellata: Benhamiidae) with additional records of earthworms from Guadeloupe (French West Indies). *Zootaxa* **4178,** 391–408 (2016).

11. Pavlicek, T. & Csuzdi, C. Earthworm fauna of French Guiana. *Adv. 5th Int. Oligochaeta Taxon. Meet.* **7140,** 107–110 (2012).

12. Brown, G. G. *et al.* Exotic, peregrine, and invasive earthworms in Brazil: Diversity, distribution, and effects on soils and plants. *Caribb. J. Sci.* **42,** 339–358 (2006).

13. Taheri, S. *et al.* Complex taxonomy of the 'brush tail' peregrine earthworm Pontoscolex corethrurus. *Mol. Phylogenet. Evol.* **124,** 60–70 (2018).

14. Köhler, F., Criscione, F., Burghardt, I. & Kessner, V. The Enidae of Timor (Stylommatophora: Orthurethra). *Molluscan Res.* **5818,** (2016).

15. Rheims, C. A. Caayguara, a new genus of huntsman spiders from the Brazilian Atlantic Forest (Araneae: Sparassidae). *Zootaxa* 1–29 (2010). doi:10.5281/zenodo.198317

16. Araujo da Silva, L. P. Modelagem de distribuição de Pickeliana pickeli (Opiliones: Laniatores: Stygnidae), seu uso na Biogeografia Histórica da Mata Atlântica e na inferência de modos de especiação. (UNIVERSIDADE FEDERAL DA PARAÍBA CENTRO, 2013).

17. Carranza, S., Baguñà, J. & Riutort, M. Origin and evolution of paralogous rRNA gene clusters within the flatworm family Dugesiidae (Platyhelminthes, Tricladida). *J. Mol. Evol.* **49,** 250–259 (1999).

18. Kury, A. B. Early lineages of Gonyleptidae (Arachnida Opiliones Laniatores). *Trop. Zool.* **7,** 343–353 (1994).

19. Bragagnolo, C. & Pinto-Da-Rocha, R. Systematic review of Promitobates Roewer, 1913 and cladistic analysis of Mitobatinae Simon, 1879 (Arachnida: Opiliones: Gonyleptidae). *Zootaxa* **1879,** 1–48 (2012).

20. Amédégnato, C. & Devriese, H. Global diversity of true and pygmy grasshoppers (Acridomorpha, Orthoptera) in freshwater. *Hydrobiologia* **595,** 535–543 (2008).

Supplementary Figures

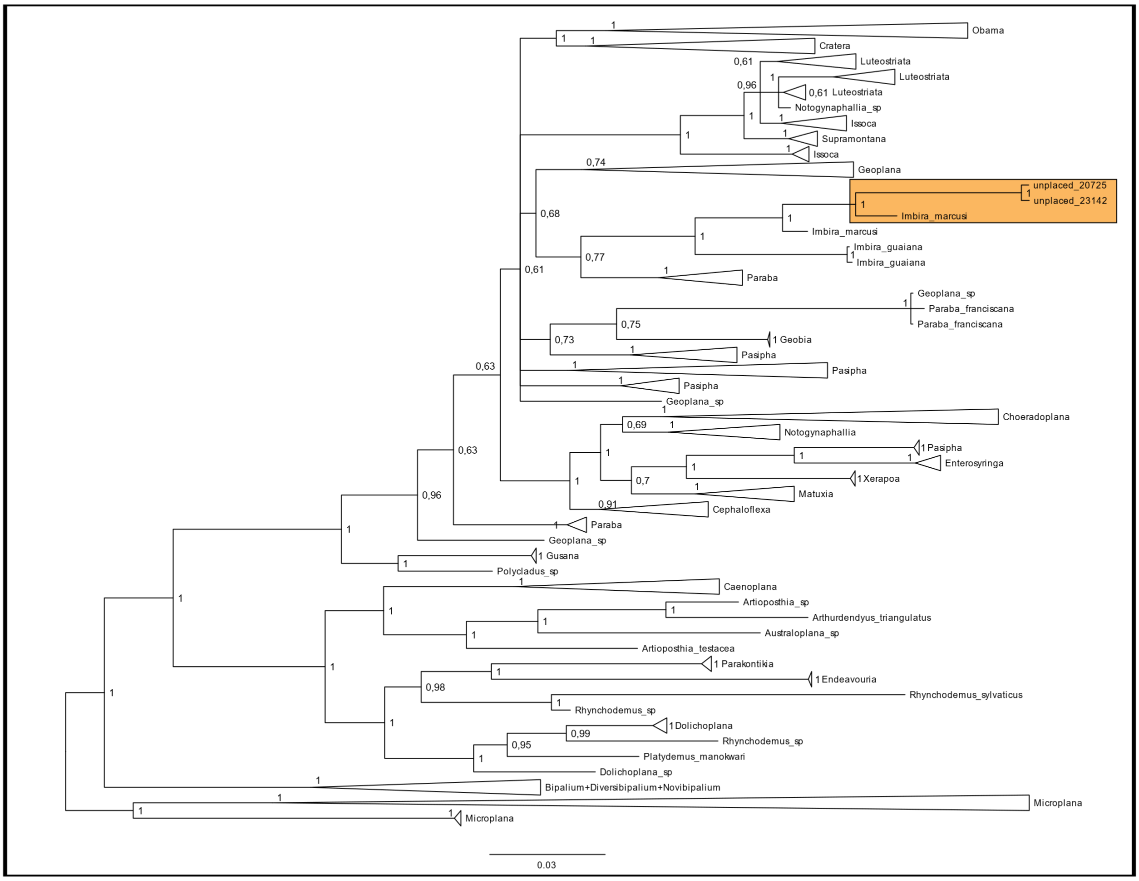

Figure S1. MAP tree of "unplaced_23142" from CbCubatao and "unplaced_20725" from CbSantoA. The query sequences are placed in a clade with a sequence of *I. marcusi* with a PP=1.
